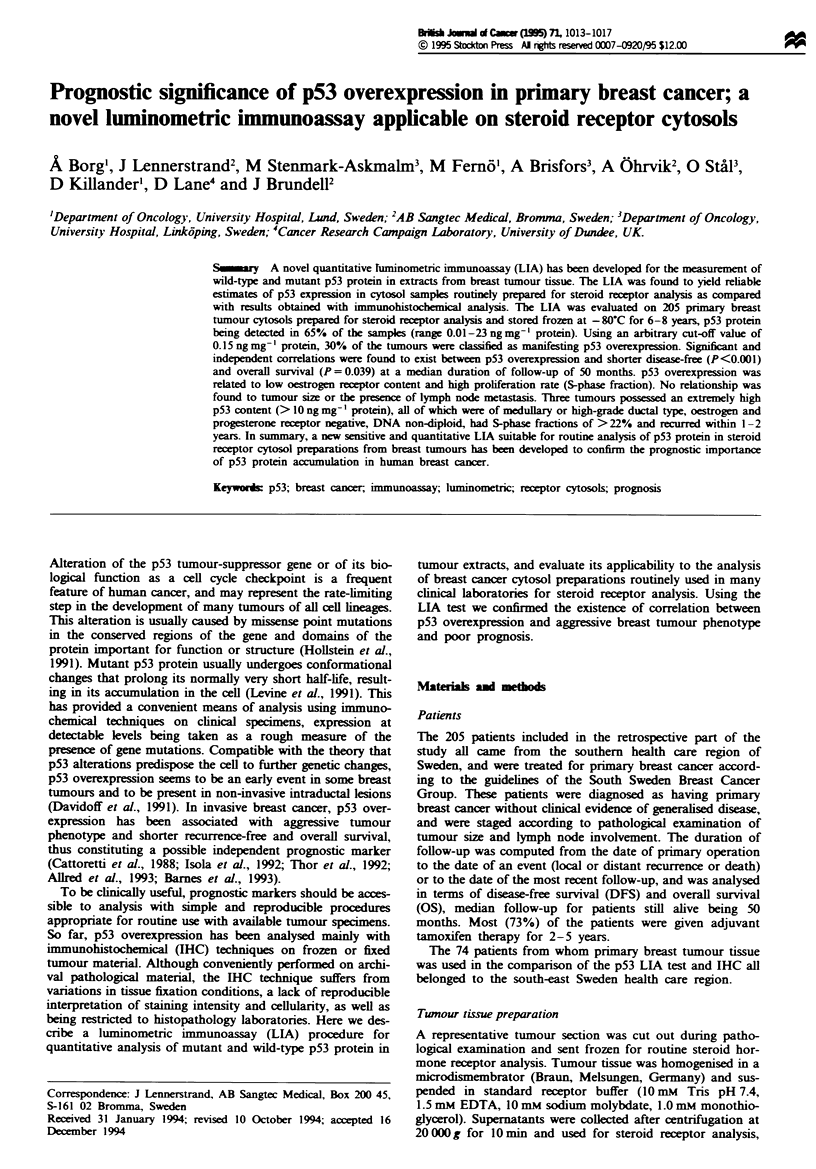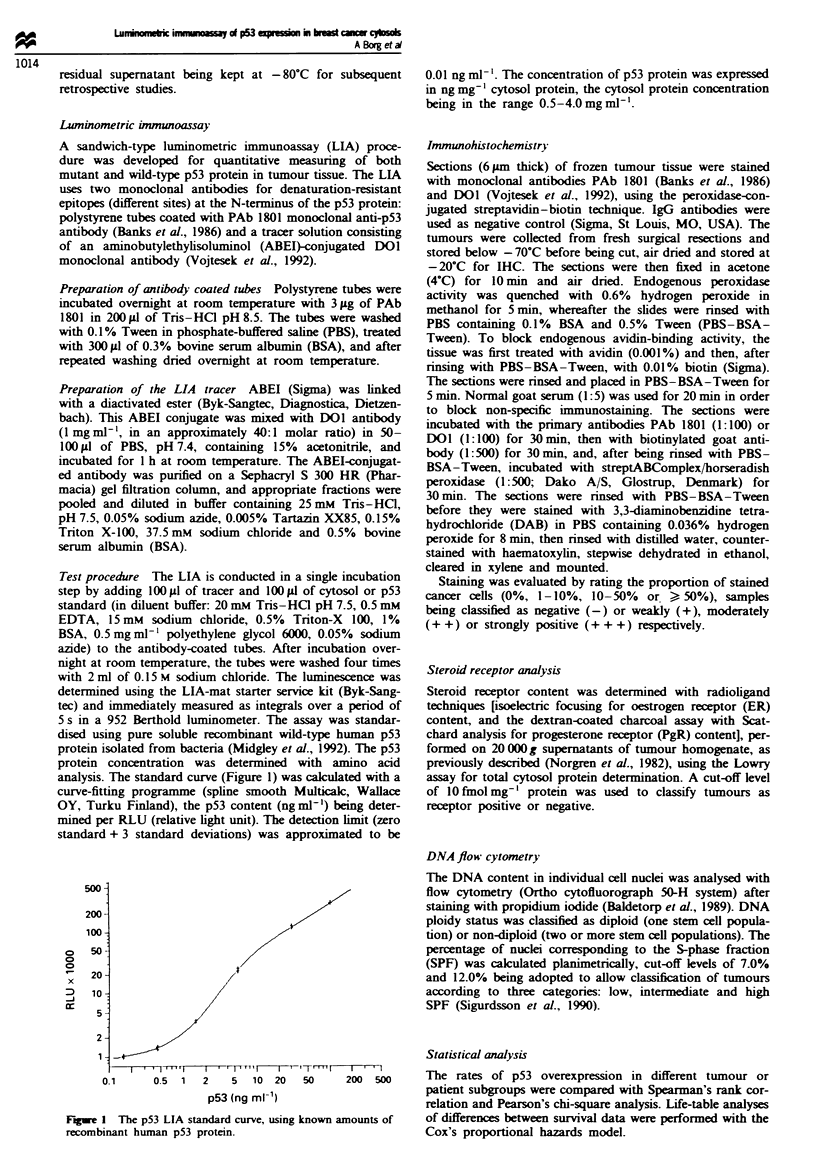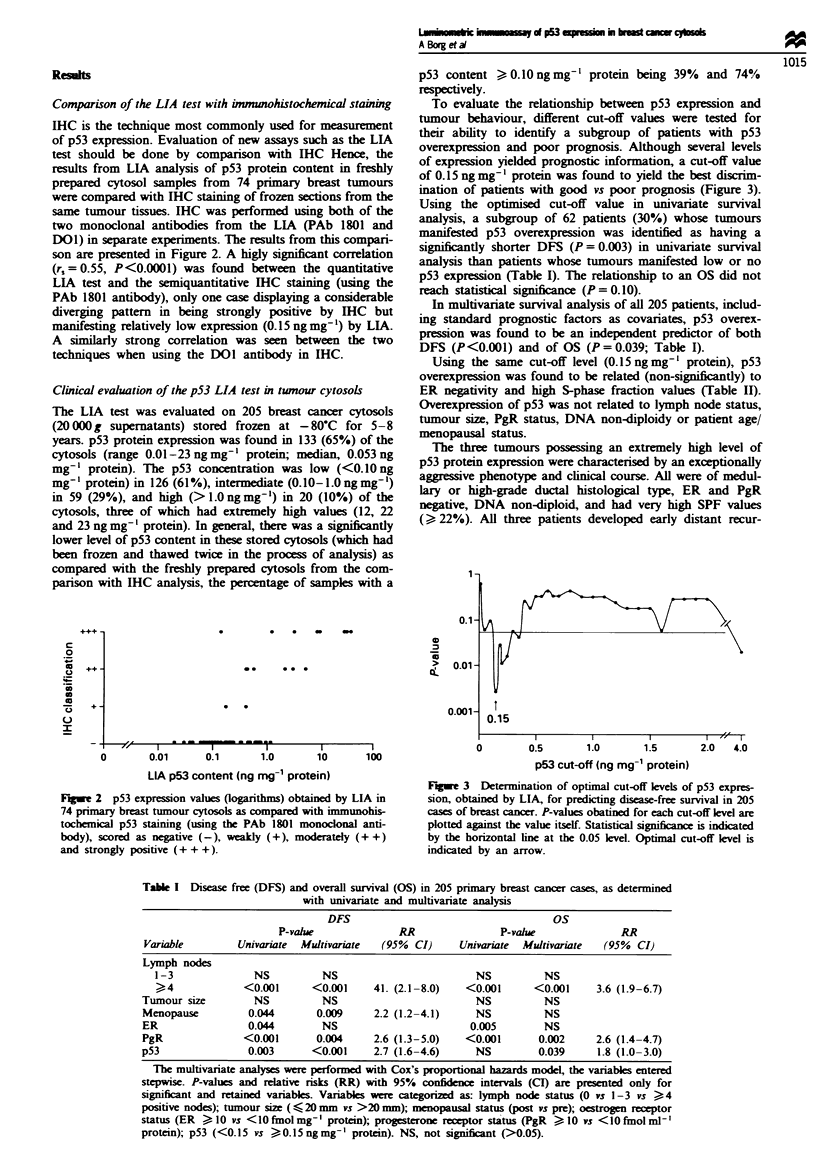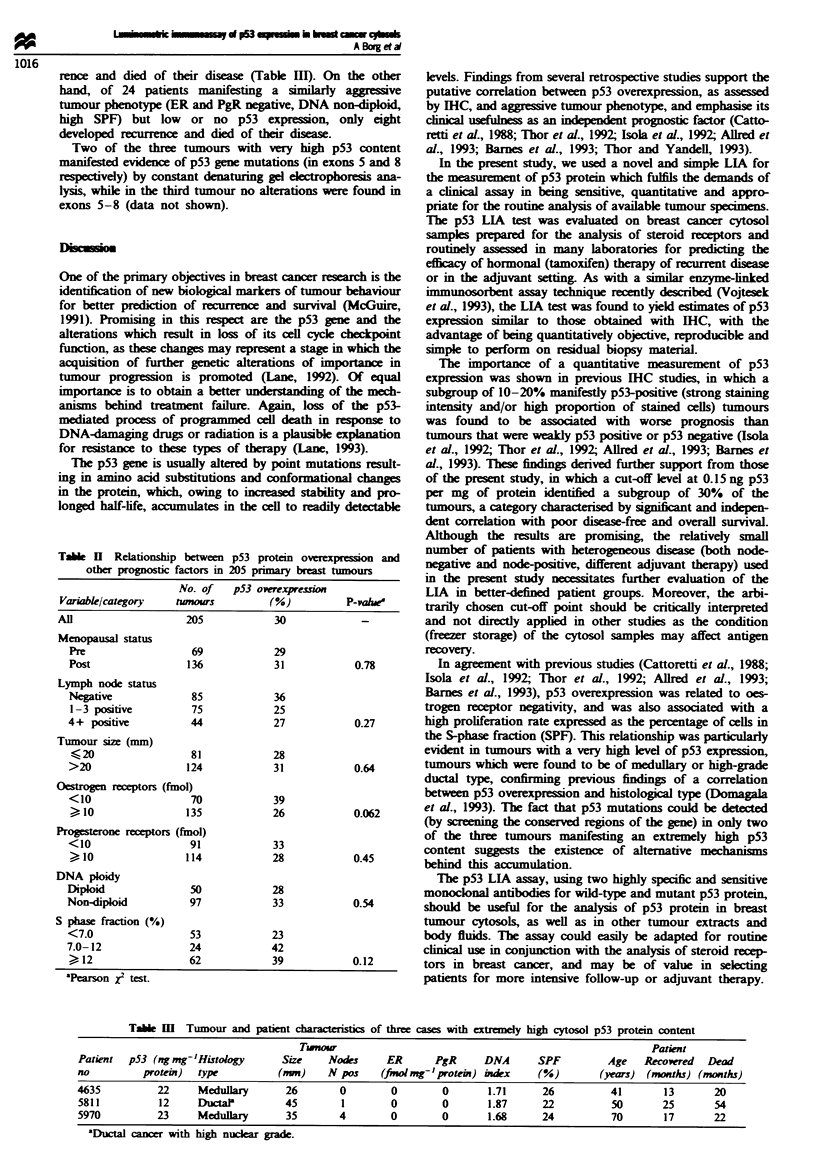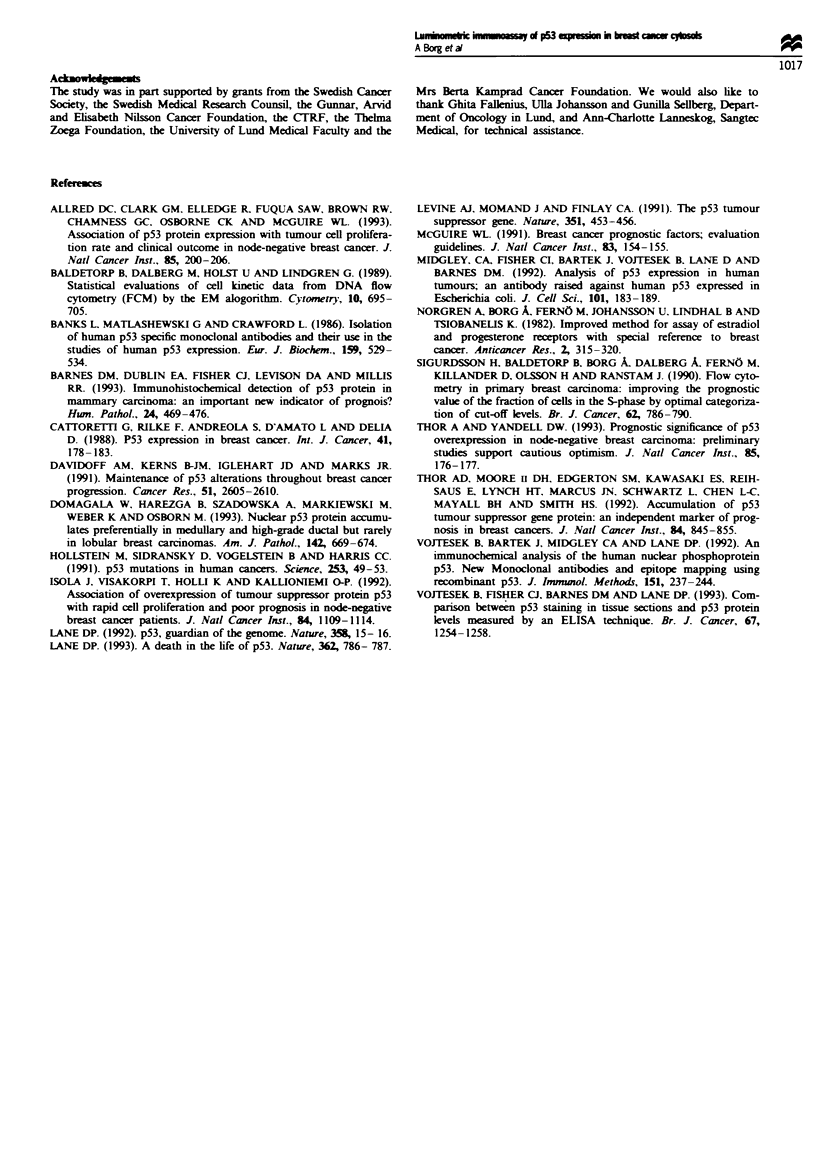# Prognostic significance of p53 overexpression in primary breast cancer; a novel luminometric immunoassay applicable on steroid receptor cytosols.

**DOI:** 10.1038/bjc.1995.195

**Published:** 1995-05

**Authors:** A. Borg, J. Lennerstrand, M. Stenmark-Askmalm, M. Fernö, A. Brisfors, A. Ohrvik, O. Stål, D. Killander, D. Lane, J. Brundell

**Affiliations:** Department of Oncology, University Hospital, Lund, Sweden.

## Abstract

A novel quantitative luminometric immunoassay (LIA) has been developed for the measurement of wild-type and mutant p53 protein in extracts from breast tumour tissue. The LIA was found to yield reliable estimates of p53 expression in cytosol samples routinely prepared for steroid receptor analysis as compared with results obtained with immunohistochemical analysis. The LIA was evaluated on 205 primary breast tumour cytosols prepared for steroid receptor analysis and stored frozen at -80 degrees C for 6-8 years, p53 protein being detected in 65% of the samples (range 0.01-23 ng mg-1 protein). Using an arbitrary cut-off value of 0.15 ng mg-1 protein, 30% of the tumours were classified as manifesting p53 overexpression. Significant and independent correlations were found to exist between p53 overexpression and shorter disease-free (P < 0.001) and overall survival (P = 0.039) at a median duration of follow-up of 50 months. p53 overexpression was related to low oestrogen receptor content and high proliferation rate (S-phase fraction). No relationship was found to tumour size or the presence of lymph node metastasis. Three tumours possessed an extremely high p53 content (> 10 ng mg-1 protein), all of which were of medullary or high-grade ductal type, oestrogen and progesterone receptor negative, DNA non-diploid, had S-phase fractions of > 22% and recurred within 1-2 years. In summary, a new sensitive and quantitative LIA suitable for routine analysis of p53 protein in steroid receptor cytosol preparations from breast tumours has been developed to confirm the prognostic importance of p53 protein accumulation in human breast cancer.


British Journal of Cancer (1995) 71, 1013–1017

© 1995 Stockton Press All rights reserved 0007–0920/95 $12.00

### Prognostic significance of p53 overexpression in primary breast cancer; a novel luminometric immunoassay applicable on steroid receptor cytosols

Å Borg[1], J Lennerstrand[2], M Stenmark-Askmalm[3], M Fernö[1], A Brisfors[3], A Öhrvik[2], O Stål[3], D Killander[1], D Lane[4] and J Brundell[2]

[1]*Department of Oncology, University Hospital, Lund, Sweden;* [2]*AB Sangtec Medical, Bromma, Sweden;* [3]*Department of Oncology, University Hospital, Linköping, Sweden;* [4]*Cancer Research Campaign Laboratory, University of Dundee, UK.*

**Summary** A novel quantitative luminometric immunoassay (LIA) has been developed for the measurement of wild-type and mutant p53 protein in extracts from breast tumour tissue. The LIA was found to yield reliable estimates of p53 expression in cytosol samples routinely prepared for steroid receptor analysis as compared with results obtained with immunohistochemical analysis. The LIA was evaluated on 205 primary breast tumour cytosols prepared for steroid receptor analysis and stored frozen at −80°C for 6–8 years, p53 protein being detected in 65% of the samples (range 0.01–23 ng mg$^{-1}$ protein). Using an arbitrary cut-off value of 0.15 ng mg$^{-1}$ protein, 30% of the tumours were classified as manifesting p53 overexpression. Significant and independent correlations were found to exist between p53 overexpression and shorter disease-free ($P<0.001$) and overall survival ($P=0.039$) at a median duration of follow-up of 50 months. p53 overexpression was related to low oestrogen receptor content and high proliferation rate (S-phase fraction). No relationship was found to tumour size or the presence of lymph node metastasis. Three tumours possessed an extremely high p53 content (>10 ng mg$^{-1}$ protein), all of which were of medullary or high-grade ductal type, oestrogen and progesterone receptor negative, DNA non-diploid, had S-phase fractions of >22% and recurred within 1–2 years. In summary, a new sensitive and quantitative LIA suitable for routine analysis of p53 protein in steroid receptor cytosol preparations from breast tumours has been developed to confirm the prognostic importance of p53 protein accumulation in human breast cancer.

**Keywords:** p53; breast cancer; immunoassay; luminometric; receptor cytosols; prognosis

Alteration of the p53 tumour-suppressor gene or of its biological function as a cell cycle checkpoint is a frequent feature of human cancer, and may represent the rate-limiting step in the development of many tumours of all cell lineages. This alteration is usually caused by missense point mutations in the conserved regions of the gene and domains of the protein important for function or structure (Hollstein *et al.*, 1991). Mutant p53 protein usually undergoes conformational changes that prolong its normally very short half-life, resulting in its accumulation in the cell (Levine *et al.*, 1991). This has provided a convenient means of analysis using immunochemical techniques on clinical specimens, expression at detectable levels being taken as a rough measure of the presence of gene mutations. Compatible with the theory that p53 alterations predispose the cell to further genetic changes, p53 overexpression seems to be an early event in some breast tumours and to be present in non-invasive intraductal lesions (Davidoff *et al.*, 1991). In invasive breast cancer, p53 overexpression has been associated with aggressive tumour phenotype and shorter recurrence-free and overall survival, thus constituting a possible independent prognostic marker (Cattoretti *et al.*, 1988; Isola *et al.*, 1992; Thor *et al.*, 1992; Allred *et al.*, 1993; Barnes *et al.*, 1993).

To be clinically useful, prognostic markers should be accessible to analysis with simple and reproducible procedures appropriate for routine use with available tumour specimens. So far, p53 overexpression has been analysed mainly with immunohistochemical (IHC) techniques on frozen or fixed tumour material. Although conveniently performed on archival pathological material, the IHC technique suffers from variations in tissue fixation conditions, a lack of reproducible interpretation of staining intensity and cellularity, as well as being restricted to histopathology laboratories. Here we describe a luminometric immunoassay (LIA) procedure for quantitative analysis of mutant and wild-type p53 protein in tumour extracts, and evaluate its applicability to the analysis of breast cancer cytosol preparations routinely used in many clinical laboratories for steroid receptor analysis. Using the LIA test we confirmed the existence of correlation between p53 overexpression and aggressive breast tumour phenotype and poor prognosis.

### Materials and methods

### *Patients*

The 205 patients included in the retrospective part of the study all came from the southern health care region of Sweden, and were treated for primary breast cancer according to the guidelines of the South Sweden Breast Cancer Group. These patients were diagnosed as having primary breast cancer without clinical evidence of generalised disease, and were staged according to pathological examination of tumour size and lymph node involvement. The duration of follow-up was computed from the date of primary operation to the date of an event (local or distant recurrence or death) or to the date of the most recent follow-up, and was analysed in terms of disease-free survival (DFS) and overall survival (OS), median follow-up for patients still alive being 50 months. Most (73%) of the patients were given adjuvant tamoxifen therapy for 2–5 years.

The 74 patients from whom primary breast tumour tissue was used in the comparison of the p53 LIA test and IHC all belonged to the south-east Sweden health care region.

### *Tumour tissue preparation*

A representative tumour section was cut out during pathological examination and sent frozen for routine steroid hormone receptor analysis. Tumour tissue was homogenised in a microdismembrator (Braun, Melsungen, Germany) and suspended in standard receptor buffer (10 mM Tris pH 7.4, 1.5 mM EDTA, 10 mM sodium molybdate, 1.0 mM monothioglycerol). Supernatants were collected after centrifugation at 20 000 *g* for 10 min and used for steroid receptor analysis,

Correspondence: J Lennerstrand, AB Sangtec Medical, Box 200 45, S-161 02 Bromma, Sweden
Received 31 January 1994; revised 10 October 1994; accepted 16 December 1994

residual supernatant being kept at −80°C for subsequent retrospective studies.

*Luminometric immunoassay*

A sandwich-type luminometric immunoassay (LIA) procedure was developed for quantitative measuring of both mutant and wild-type p53 protein in tumour tissue. The LIA uses two monoclonal antibodies for denaturation-resistant epitopes (different sites) at the N-terminus of the p53 protein: polystyrene tubes coated with PAb 1801 monoclonal anti-p53 antibody (Banks *et al.*, 1986) and a tracer solution consisting of an aminobutylethylisoluminol (ABEI)-conjugated DO1 monoclonal antibody (Vojtesek *et al.*, 1992).

*Preparation of antibody coated tubes* Polystyrene tubes were incubated overnight at room temperature with 3 μg of PAb 1801 in 200 μl of Tris–HCl pH 8.5. The tubes were washed with 0.1% Tween in phosphate-buffered saline (PBS), treated with 300 μl of 0.3% bovine serum albumin (BSA), and after repeated washing dried overnight at room temperature.

*Preparation of the LIA tracer* ABEI (Sigma) was linked with a diactivated ester (Byk-Sangtec, Diagnostica, Dietzenbach). This ABEI conjugate was mixed with DO1 antibody (1 mg $ml^{-1}$, in an approximately 40:1 molar ratio) in 50–100 μl of PBS, pH 7.4, containing 15% acetonitrile, and incubated for 1 h at room temperature. The ABEI-conjugated antibody was purified on a Sephacryl S 300 HR (Pharmacia) gel filtration column, and appropriate fractions were pooled and diluted in buffer containing 25 mM Tris–HCl, pH 7.5, 0.05% sodium azide, 0.005% Tartazin XX85, 0.15% Triton X-100, 37.5 mM sodium chloride and 0.5% bovine serum albumin (BSA).

*Test procedure* The LIA is conducted in a single incubation step by adding 100 μl of tracer and 100 μl of cytosol or p53 standard (in diluent buffer: 20 mM Tris–HCl pH 7.5, 0.5 mM EDTA, 15 mM sodium chloride, 0.5% Triton-X 100, 1% BSA, 0.5 mg $ml^{-1}$ polyethylene glycol 6000, 0.05% sodium azide) to the antibody-coated tubes. After incubation overnight at room temperature, the tubes were washed four times with 2 ml of 0.15 M sodium chloride. The luminescence was determined using the LIA-mat starter service kit (Byk-Sangtec) and immediately measured as integrals over a period of 5 s in a 952 Berthold luminometer. The assay was standardised using pure soluble recombinant wild-type human p53 protein isolated from bacteria (Midgley *et al.*, 1992). The p53 protein concentration was determined with amino acid analysis. The standard curve (Figure 1) was calculated with a curve-fitting programme (spline smooth Multicalc, Wallace OY, Turku Finland), the p53 content (ng $ml^{-1}$) being determined per RLU (relative light unit). The detection limit (zero standard + 3 standard deviations) was approximated to be 0.01 ng $ml^{-1}$. The concentration of p53 protein was expressed in ng $mg^{-1}$ cytosol protein, the cytosol protein concentration being in the range 0.5–4.0 mg $ml^{-1}$.

**Figure 1** The p53 LIA standard curve, using known amounts of recombinant human p53 protein.

*Immunohistochemistry*

Sections (6 μm thick) of frozen tumour tissue were stained with monoclonal antibodies PAb 1801 (Banks *et al.*, 1986) and DO1 (Vojtesek *et al.*, 1992), using the peroxidase-conjugated streptavidin–biotin technique. IgG antibodies were used as negative control (Sigma, St Louis, MO, USA). The tumours were collected from fresh surgical resections and stored below −70°C before being cut, air dried and stored at −20°C for IHC. The sections were then fixed in acetone (4°C) for 10 min and air dried. Endogenous peroxidase activity was quenched with 0.6% hydrogen peroxide in methanol for 5 min, whereafter the slides were rinsed with PBS containing 0.1% BSA and 0.5% Tween (PBS–BSA–Tween). To block endogenous avidin-binding activity, the tissue was first treated with avidin (0.001%) and then, after rinsing with PBS–BSA–Tween, with 0.01% biotin (Sigma). The sections were rinsed and placed in PBS–BSA–Tween for 5 min. Normal goat serum (1:5) was used for 20 min in order to block non-specific immunostaining. The sections were incubated with the primary antibodies PAb 1801 (1:100) or DO1 (1:100) for 30 min, then with biotinylated goat antibody (1:500) for 30 min, and, after being rinsed with PBS–BSA–Tween, incubated with streptABComplex/horseradish peroxidase (1:500; Dako A/S, Glostrup, Denmark) for 30 min. The sections were rinsed with PBS–BSA–Tween before they were stained with 3,3-diaminobenzidine tetrahydrochloride (DAB) in PBS containing 0.036% hydrogen peroxide for 8 min, then rinsed with distilled water, counterstained with haematoxylin, stepwise dehydrated in ethanol, cleared in xylene and mounted.

Staining was evaluated by rating the proportion of stained cancer cells (0%, 1–10%, 10–50% or ⩾50%), samples being classified as negative (−) or weakly (+), moderately (+ +) or strongly positive (+ + +) respectively.

*Steroid receptor analysis*

Steroid receptor content was determined with radioligand techniques [isoelectric focusing for oestrogen receptor (ER) content, and the dextran-coated charcoal assay with Scatchard analysis for progesterone receptor (PgR) content], performed on 20 000 *g* supernatants of tumour homogenate, as previously described (Norgren *et al.*, 1982), using the Lowry assay for total cytosol protein determination. A cut-off level of 10 fmol $mg^{-1}$ protein was used to classify tumours as receptor positive or negative.

*DNA flow cytometry*

The DNA content in individual cell nuclei was analysed with flow cytometry (Ortho cytofluorograph 50-H system) after staining with propidium iodide (Baldetorp *et al.*, 1989). DNA ploidy status was classified as diploid (one stem cell population) or non-diploid (two or more stem cell populations). The percentage of nuclei corresponding to the S-phase fraction (SPF) was calculated planimetrically, cut-off levels of 7.0% and 12.0% being adopted to allow classification of tumours according to three categories: low, intermediate and high SPF (Sigurdsson *et al.*, 1990).

*Statistical analysis*

The rates of p53 overexpression in different tumour or patient subgroups were compared with Spearman's rank correlation and Pearson's chi-square analysis. Life-table analyses of differences between survival data were performed with the Cox's proportional hazards model.

### Results

### *Comparison of the LIA test with immunohistochemical staining*

IHC is the technique most commonly used for measurement of p53 expression. Evaluation of new assays such as the LIA test should be done by comparison with IHC Hence, the results from LIA analysis of p53 protein content in freshly prepared cytosol samples from 74 primary breast tumours were compared with IHC staining of frozen sections from the same tumour tissues. IHC was performed using both of the two monoclonal antibodies from the LIA (PAb 1801 and DO1) in separate experiments. The results from this comparison are presented in Figure 2. A higly significant correlation ($r_s = 0.55$, $P < 0.0001$) was found between the quantitative LIA test and the semiquantitative IHC staining (using the PAb 1801 antibody), only one case displaying a considerable diverging pattern in being strongly positive by IHC but manifesting relatively low expression (0.15 ng mg$^{-1}$) by LIA. A similarly strong correlation was seen between the two techniques when using the DO1 antibody in IHC.

### *Clinical evaluation of the p53 LIA test in tumour cytosols*

The LIA test was evaluated on 205 breast cancer cytosols (20 000 *g* supernatants) stored frozen at −80°C for 5–8 years. p53 protein expression was found in 133 (65%) of the cytosols (range 0.01–23 ng mg$^{-1}$ protein; median, 0.053 ng mg$^{-1}$ protein). The p53 concentration was low (<0.10 ng mg$^{-1}$ protein) in 126 (61%), intermediate (0.10–1.0 ng mg$^{-1}$) in 59 (29%), and high (>1.0 ng mg$^{-1}$) in 20 (10%) of the cytosols, three of which had extremely high values (12, 22 and 23 ng mg$^{-1}$ protein). In general, there was a significantly lower level of p53 content in these stored cytosols (which had been frozen and thawed twice in the process of analysis) as compared with the freshly prepared cytosols from the comparison with IHC analysis, the percentage of samples with a p53 content ⩾0.10 ng mg$^{-1}$ protein being 39% and 74% respectively.

To evaluate the relationship between p53 expression and tumour behaviour, different cut-off values were tested for their ability to identify a subgroup of patients with p53 overexpression and poor prognosis. Although several levels of expression yielded prognostic information, a cut-off value of 0.15 ng mg$^{-1}$ protein was found to yield the best discrimination of patients with good *vs* poor prognosis (Figure 3). Using the optimised cut-off value in univariate survival analysis, a subgroup of 62 patients (30%) whose tumours manifested p53 overexpression was identified as having a significantly shorter DFS ($P = 0.003$) in univariate survival analysis than patients whose tumours manifested low or no p53 expression (Table I). The relationship to an OS did not reach statistical significance ($P = 0.10$).

In multivariate survival analysis of all 205 patients, including standard prognostic factors as covariates, p53 overexpression was found to be an independent predictor of both DFS ($P < 0.001$) and of OS ($P = 0.039$; Table I).

Using the same cut-off level (0.15 ng mg$^{-1}$ protein), p53 overexpression was found to be related (non-significantly) to ER negativity and high S-phase fraction values (Table II). Overexpression of p53 was not related to lymph node status, tumour size, PgR status, DNA non-diploidy or patient age/menopausal status.

The three tumours possessing an extremely high level of p53 protein expression were characterised by an exceptionally aggressive phenotype and clinical course. All were of medullary or high-grade ductal histological type, ER and PgR negative, DNA non-diploid, and had very high SPF values (⩾22%). All three patients developed early distant recur-

**Figure 2** p53 expression values (logarithms) obtained by LIA in 74 primary breast tumour cytosols as compared with immunohistochemical p53 staining (using the PAb 1801 monoclonal antibody), scored as negative (−), weakly (+), moderately (++) and strongly positive (+++).

**Figure 3** Determination of optimal cut-off levels of p53 expression, obtained by LIA, for predicting disease-free survival in 205 cases of breast cancer. *P*-values obatined for each cut-off level are plotted against the value itself. Statistical significance is indicated by the horizontal line at the 0.05 level. Optimal cut-off level is indicated by an arrow.

**Table I** Disease free (DFS) and overall survival (OS) in 205 primary breast cancer cases, as determined with univariate and multivariate analysis

| | *DFS* | | | *OS* | | |
|---|---|---|---|---|---|---|
| | P-*value* | | *RR* | P-*value* | | *RR* |
| *Variable* | *Univariate* | *Multivariate* | *(95% CI)* | *Univariate* | *Multivariate* | *(95% CI)* |
| Lymph nodes | | | | | | |
| 1–3 | NS | NS | | NS | NS | |
| ⩾4 | <0.001 | <0.001 | 41. (2.1–8.0) | <0.001 | <0.001 | 3.6 (1.9–6.7) |
| Tumour size | NS | NS | | NS | NS | |
| Menopause | 0.044 | 0.009 | 2.2 (1.2–4.1) | NS | NS | |
| ER | 0.044 | NS | | 0.005 | NS | |
| PgR | <0.001 | 0.004 | 2.6 (1.3–5.0) | <0.001 | 0.002 | 2.6 (1.4–4.7) |
| p53 | 0.003 | <0.001 | 2.7 (1.6–4.6) | NS | 0.039 | 1.8 (1.0–3.0) |

The multivariate analyses were performed with Cox's proportional hazards model, the variables entered stepwise. *P*-values and relative risks (RR) with 95% confidence intervals (CI) are presented only for significant and retained variables. Variables were categorized as: lymph node status (0 *vs* 1–3 *vs* ⩾4 positive nodes); tumour size (⩽20 mm *vs* >20 mm); menopausal status (post *vs* pre); oestrogen receptor status (ER ⩾10 *vs* <10 fmol mg$^{-1}$ protein); progesterone receptor status (PgR ⩾10 *vs* <10 fmol ml$^{-1}$ protein); p53 (<0.15 *vs* ⩾0.15 ng mg$^{-1}$ protein). NS, not significant (>0.05).

rence and died of their disease (Table III). On the other hand, of 24 patients manifesting a similarly aggressive tumour phenotype (ER and PgR negative, DNA non-diploid, high SPF) but low or no p53 expression, only eight developed recurrence and died of their disease.

Two of the three tumours with very high p53 content manifested evidence of p53 gene mutations (in exons 5 and 8 respectively) by constant denaturing gel electrophoresis analysis, while in the third tumour no alterations were found in exons 5-8 (data not shown).

### Discussion

One of the primary objectives in breast cancer research is the identification of new biological markers of tumour behaviour for better prediction of recurrence and survival (McGuire, 1991). Promising in this respect are the p53 gene and the alterations which result in loss of its cell cycle checkpoint function, as these changes may represent a stage in which the acquisition of further genetic alterations of importance in tumour progression is promoted (Lane, 1992). Of equal importance is to obtain a better understanding of the mechanisms behind treatment failure. Again, loss of the p53-mediated process of programmed cell death in response to DNA-damaging drugs or radiation is a plausible explanation for resistance to these types of therapy (Lane, 1993).

The p53 gene is usually altered by point mutations resulting in amino acid substitutions and conformational changes in the protein, which, owing to increased stability and prolonged half-life, accumulates in the cell to readily detectable levels. Findings from several retrospective studies support the putative correlation between p53 overexpression, as assessed by IHC, and aggressive tumour phenotype, and emphasise its clinical usefulness as an independent prognostic factor (Cattoretti *et al.*, 1988; Thor *et al.*, 1992; Isola *et al.*, 1992; Allred *et al.*, 1993; Barnes *et al.*, 1993; Thor and Yandell, 1993).

In the present study, we used a novel and simple LIA for the measurement of p53 protein which fulfils the demands of a clinical assay in being sensitive, quantitative and appropriate for the routine analysis of available tumour specimens. The p53 LIA test was evaluated on breast cancer cytosol samples prepared for the analysis of steroid receptors and routinely assessed in many laboratories for predicting the efficacy of hormonal (tamoxifen) therapy of recurrent disease or in the adjuvant setting. As with a similar enzyme-linked immunosorbent assay technique recently described (Vojtesek *et al.*, 1993), the LIA test was found to yield estimates of p53 expression similar to those obtained with IHC, with the advantage of being quantitatively objective, reproducible and simple to perform on residual biopsy material.

The importance of a quantitative measurement of p53 expression was shown in previous IHC studies, in which a subgroup of 10-20% manifestly p53-positive (strong staining intensity and/or high proportion of stained cells) tumours was found to be associated with worse prognosis than tumours that were weakly p53 positive or p53 negative (Isola *et al.*, 1992; Thor *et al.*, 1992; Allred *et al.*, 1993; Barnes *et al.*, 1993). These findings derived further support from those of the present study, in which a cut-off level at 0.15 ng p53 per mg of protein identified a subgroup of 30% of the tumours, a category characterised by significant and independent correlation with poor disease-free and overall survival. Although the results are promising, the relatively small number of patients with heterogeneous disease (both node-negative and node-positive, different adjuvant therapy) used in the present study necessitates further evaluation of the LIA in better-defined patient groups. Moreover, the arbitrarily chosen cut-off point should be critically interpreted and not directly applied in other studies as the condition (freezer storage) of the cytosol samples may affect antigen recovery.

In agreement with previous studies (Cattoretti *et al.*, 1988; Isola *et al.*, 1992; Thor *et al.*, 1992; Allred *et al.*, 1993; Barnes *et al.*, 1993), p53 overexpression was related to oestrogen receptor negativity, and was also associated with a high proliferation rate expressed as the percentage of cells in the S-phase fraction (SPF). This relationship was particularly evident in tumours with a very high level of p53 expression, tumours which were found to be of medullary or high-grade ductal type, confirming previous findings of a correlation between p53 overexpression and histological type (Domagala *et al.*, 1993). The fact that p53 mutations could be detected (by screening the conserved regions of the gene) in only two of the three tumours manifesting an extremely high p53 content suggests the existence of alternative mechanisms behind this accumulation.

The p53 LIA assay, using two highly specific and sensitive monoclonal antibodies for wild-type and mutant p53 protein, should be useful for the analysis of p53 protein in breast tumour cytosols, as well as in other tumour extracts and body fluids. The assay could easily be adapted for routine clinical use in conjunction with the analysis of steroid receptors in breast cancer, and may be of value in selecting patients for more intensive follow-up or adjuvant therapy.

**Table II** Relationship between p53 protein overexpression and other prognostic factors in 205 primary breast tumours

| *Variable/category* | *No. of tumours* | *p53 overexpression (%)* | *P-value*[a] |
|---|---|---|---|
| All | 205 | 30 | – |
| Menopausal status | | | |
| Pre | 69 | 29 | |
| Post | 136 | 31 | 0.78 |
| Lymph node status | | | |
| Negative | 85 | 36 | |
| 1-3 positive | 75 | 25 | |
| 4+ positive | 44 | 27 | 0.27 |
| Tumour size (mm) | | | |
| ⩽20 | 81 | 28 | |
| >20 | 124 | 31 | 0.64 |
| Oestrogen receptors (fmol) | | | |
| <10 | 70 | 39 | |
| ⩾10 | 135 | 26 | 0.062 |
| Progesterone receptors (fmol) | | | |
| <10 | 91 | 33 | |
| ⩾10 | 114 | 28 | 0.45 |
| DNA ploidy | | | |
| Diploid | 50 | 28 | |
| Non-diploid | 97 | 33 | 0.54 |
| S phase fraction (%) | | | |
| <7.0 | 53 | 23 | |
| 7.0-12 | 24 | 42 | |
| ⩾12 | 62 | 39 | 0.12 |

[a]Pearson $\chi^2$ test.

**Table III** Tumour and patient characteristics of three cases with extremely high cytosol p53 protein content

| *Patient no* | *p53 (ng mg⁻¹ protein)* | *Histology type* | *Tumour Size (mm)* | *Tumour Nodes N pos* | *ER (fmol mg⁻¹ protein)* | *PgR (fmol mg⁻¹ protein)* | *DNA index* | *SPF (%)* | *Patient Age (years)* | *Patient Recovered (months)* | *Patient Dead (months)* |
|---|---|---|---|---|---|---|---|---|---|---|---|
| 4635 | 22 | Medullary | 26 | 0 | 0 | 0 | 1.71 | 26 | 41 | 13 | 20 |
| 5811 | 12 | Ductal[a] | 45 | 1 | 0 | 0 | 1.87 | 22 | 50 | 25 | 54 |
| 5970 | 23 | Medullary | 35 | 4 | 0 | 0 | 1.68 | 24 | 70 | 17 | 22 |

[a]Ductal cancer with high nuclear grade.

**Acknowledgements**

The study was in part supported by grants from the Swedish Cancer Society, the Swedish Medical Research Counsil, the Gunnar, Arvid and Elisabeth Nilsson Cancer Foundation, the CTRF, the Thelma Zoega Foundation, the University of Lund Medical Faculty and the Mrs Berta Kamprad Cancer Foundation. We would also like to thank Ghita Fallenius, Ulla Johansson and Gunilla Sellberg, Department of Oncology in Lund, and Ann-Charlotte Lanneskog, Sangtec Medical, for technical assistance.

**References**

ALLRED DC, CLARK GM, ELLEDGE R, FUQUA SAW, BROWN RW, CHAMNESS GC, OSBORNE CK AND McGUIRE WL. (1993). Association of p53 protein expression with tumour cell proliferation rate and clinical outcome in node-negative breast cancer. *J. Natl Cancer Inst.*, **85**, 200-206.

BALDETORP B, DALBERG M, HOLST U AND LINDGREN G. (1989). Statistical evaluations of cell kinetic data from DNA flow cytometry (FCM) by the EM alogorithm. *Cytometry*, **10**, 695-705.

BANKS L, MATLASHEWSKI G AND CRAWFORD L. (1986). Isolation of human p53 specific monoclonal antibodies and their use in the studies of human p53 expression. *Eur. J. Biochem.*, **159**, 529-534.

BARNES DM, DUBLIN EA, FISHER CJ, LEVISON DA AND MILLIS RR. (1993). Immunohistochemical detection of p53 protein in mammary carcinoma: an important new indicator of prognois? *Hum. Pathol.*, **24**, 469-476.

CATTORETTI G, RILKE F, ANDREOLA S, D'AMATO L AND DELIA D. (1988). P53 expression in breast cancer. *Int. J. Cancer*, **41**, 178-183.

DAVIDOFF AM, KERNS B-JM, IGLEHART JD AND MARKS JR. (1991). Maintenance of p53 alterations throughout breast cancer progression. *Cancer Res.*, **51**, 2605-2610.

DOMAGALA W, HAREZGA B, SZADOWSKA A, MARKIEWSKI M, WEBER K AND OSBORN M. (1993). Nuclear p53 protein accumulates preferentially in medullary and high-grade ductal but rarely in lobular breast carcinomas. *Am. J. Pathol.*, **142**, 669-674.

HOLLSTEIN M, SIDRANSKY D, VOGELSTEIN B AND HARRIS CC. (1991). p53 mutations in human cancers. *Science*, **253**, 49-53.

ISOLA J, VISAKORPI T, HOLLI K AND KALLIONIEMI O-P. (1992). Association of overexpression of tumour suppressor protein p53 with rapid cell proliferation and poor prognosis in node-negative breast cancer patients. *J. Natl Cancer Inst.*, **84**, 1109-1114.

LANE DP. (1992). p53, guardian of the genome. *Nature*, **358**, 15- 16.

LANE DP. (1993). A death in the life of p53. *Nature*, **362**, 786- 787.

LEVINE AJ, MOMAND J AND FINLAY CA. (1991). The p53 tumour suppressor gene. *Nature*, **351**, 453-456.

McGUIRE WL. (1991). Breast cancer prognostic factors; evaluation guidelines. *J. Natl Cancer Inst.*, **83**, 154-155.

MIDGLEY, CA, FISHER CI, BARTEK J, VOJTESEK B, LANE D AND BARNES DM. (1992). Analysis of p53 expression in human tumours; an antibody raised against human p53 expressed in Escherichia coli. *J. Cell Sci.*, **101**, 183-189.

NORGREN A, BORG Å, FERNÖ M, JOHANSSON U, LINDHAL B AND TSIOBANELIS K. (1982). Improved method for assay of estradiol and progesterone receptors with special reference to breast cancer. *Anticancer Res.*, **2**, 315-320.

SIGURDSSON H, BALDETORP B, BORG Å, DALBERG Å, FERNÖ M, KILLANDER D, OLSSON H AND RANSTAM J. (1990). Flow cytometry in primary breast carcinoma: improving the prognostic value of the fraction of cells in the S-phase by optimal categorization of cut-off levels. *Br. J. Cancer*, **62**, 786-790.

THOR A AND YANDELL DW. (1993). Prognostic significance of p53 overexpression in node-negative breast carcinoma: preliminary studies support cautious optimism. *J. Natl Cancer Inst.*, **85**, 176-177.

THOR AD, MOORE II DH, EDGERTON SM, KAWASAKI ES, REIHSAUS E, LYNCH HT, MARCUS JN, SCHWARTZ L, CHEN L-C, MAYALL BH AND SMITH HS. (1992). Accumulation of p53 tumour suppressor gene protein: an independent marker of prognosis in breast cancers. *J. Natl Cancer Inst.*, **84**, 845-855.

VOJTESEK B, BARTEK J, MIDGLEY CA AND LANE DP. (1992). An immunochemical analysis of the human nuclear phosphoprotein p53. New Monoclonal antibodies and epitope mapping using recombinant p53. *J. Immunol. Methods*, **151**, 237-244.

VOJTESEK B, FISHER CJ, BARNES DM AND LANE DP. (1993). Comparison between p53 staining in tissue sections and p53 protein levels measured by an ELISA technique. *Br. J. Cancer*, **67**, 1254-1258.